# Advances in Gene Editing of Haploid Tissues in Crops

**DOI:** 10.3390/genes12091410

**Published:** 2021-09-13

**Authors:** Pankaj Bhowmik, Andriy Bilichak

**Affiliations:** 1 Aquatic and Crop Resource Development, National Research Council of Canada, Saskatoon, SK S7N 0W9, Canada; pankaj.bhowmik@nrc-cnrc.gc.ca; 2 Agriculture and Agri-Food Canada, Morden Research and Development Centre, Morden, MB R6M 1Y5, Canada

**Keywords:** doubled haploid, gene editing, CRISPR/Cas9, haploid induction

## Abstract

Emerging threats of climate change require the rapid development of improved varieties with a higher tolerance to abiotic and biotic factors. Despite the success of traditional agricultural practices, novel techniques for precise manipulation of the crop’s genome are needed. Doubled haploid (DH) methods have been used for decades in major crops to fix desired alleles in elite backgrounds in a short time. DH plants are also widely used for mapping of the quantitative trait loci (QTLs), marker-assisted selection (MAS), genomic selection (GS), and hybrid production. Recent discoveries of genes responsible for haploid induction (HI) allowed engineering this trait through gene editing (GE) in non-inducer varieties of different crops. Direct editing of gametes or haploid embryos increases GE efficiency by generating null homozygous plants following chromosome doubling. Increased understanding of the underlying genetic mechanisms responsible for spontaneous chromosome doubling in haploid plants may allow transferring this trait to different elite varieties. Overall, further improvement in the efficiency of the DH technology combined with the optimized GE could accelerate breeding efforts of the major crops.

## 1. Introduction

Gene editing (GE), through the application of designed endonucleases, rapidly advances our understanding of gene function and regulatory elements involved in gene expression, as well as allows the engineering of DNA from base pair and to the chromosome level (reviewed in [1]). However, the complexity of polyploid genomes and recalcitrance of the major staple crops to transformation prevent the rapid introduction of desired traits into elite cultivars for the generation of field-ready varieties [2]. The genomic complexity can be reduced by directly editing the haploid plant tissues generated through various techniques of haploid induction (HI). A combination of GE with HI could potentially increase throughput for the generation of improved cultivars in several crop species (e.g., *Triticum aestivum* L., *Zea mays* L., *Hordeum vulgare* L., *Brassica napus* L.). Here, we cover the most recent developments of HI engineering through GE, direct introduction of edits in haploid tissues, and use of the DH and GE technologies for hybrid breeding.

## 2. Application of Doubled Haploid Technology in Modern Breeding and Genetic Analysis

Conventional breeding of the major crops (wheat, rice, maize, etc.) is based on genetic crosses of parents with distinct traits to bring the desired alleles into a single elite variety. Fixation of target alleles usually requires 8–10 generations together with costly and laborious background screening of the large-sized populations. A low recombination rate throughout the crop’s genome or lack of it in certain regions could further exacerbate the situation due to the linkage drag effect [3,4]. Complete homozygosity at all alleles throughout the genome is possible to achieve by generating haploid lines through the process known as haploid induction (HI). Sterile haploids are then converted into fertile doubled haploid (DH) lines (*n* → 2*n*) by either natural or artificial doubling. The latter is performed by treatment with an anti-microtubule drug such as colchicine [5]. The DH technology significantly speeds up the generation of pure homozygous plants; it allows stabilizing the genetic background within two generations compared to up to eight during conventional selfing or backcrossing [6]. At the same time, linkage drag still remains an obstacle for breeding programs regardless of the method used to generate either complete homozygous or isogenic lines.

Additionally, when a single gene is introgressed, the expected probability of recovery of a homozygous plant in the F_2_ population is 1/4 [6]. With a higher number of genes, the frequency of homozygous plant recovery decreases exponentially with the formula 1/4*n*, where *n* is the number of genes that segregate independently [6]. At the same time, the frequency of haploid genotypes is 1/2*n*. Therefore, the odds of recovery of a plant homozygous for four independent genes in the selfed diploid progeny is 1/256, whereas, in the haploid population, it is 1/16. For example, the generation of hybrid crops requires an efficient male sterility system (MS). Wheat hybrid breeding programs in China increasingly rely on thermo-photo-sensitive genic male sterility (TPSGMS) [7]. The TPSGMS lines are sterile under low-temperature and short-day conditions that allow for hybrid grain production. Fertility is restored under high-temperature and long-day conditions; therefore, the system does not require a fertility restoration line and streamlines hybrid grain production. The TPSGMS trait is controlled by several recessive major genes plus several minor genes, resulting in a meagre percentage of plants being highly sterile in segregating population following a cross of sterile lines with the fertile ones. It was possible to generate only two TPSGMS lines in 14 years of conventional breeding. At the same time, the authors developed 24 elite TPSGMS lines with complete homozygosity in four years using DH techniques [7]. In addition to breeding, the HI technique benefits studying plant genetics and basic embryology [6,8]. The DH lines streamline studies of the heritability of recessive alleles and are widely used to map the quantitative trait loci (QTLs) [9].

**Figure 1:**
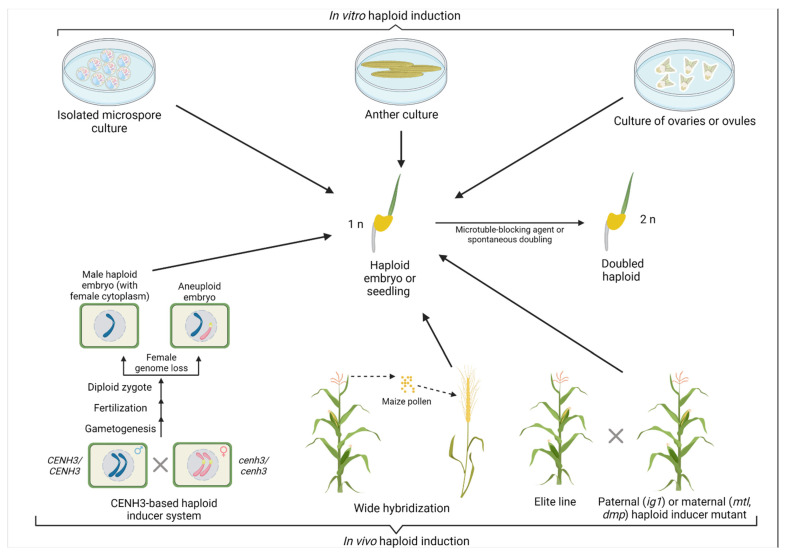
Summary of haploid induction (HI) methods. Haploids can be induced through in vitro and in vivo methods. Whereas the in vitro methods include isolated microspore culture, as well as anther and ovary/ovule culture, current in vivo methods are based on *CENH3*—mediated induction, wide hybridization techniques, and haploid inducer mutants. *CENH3*—centromere histone H3, *MTL*—*MATRILINEAL*, *DMP*—DOMAIN OF UNKNOWN FUNCTION 679 membrane protein, *ig1—indeterminate gametophyte 1*. Created with BioRender.com (website: https://biorender.com/, accessed on 29 July 2021).

Haploid plants can occur naturally, and the first such occurrence was discovered in 1922 in weed species *Datura stramonium* L. [10]. Interspecific hybridization was also found to occasionally generate haploid plants in *Nicotiana* and *Triticum compactum* [11,12]. Overall, haploids can be generated through either in vitro methods (culturing of immature male or female gametophytes) or in vivo methods (development of haploid embryo on a plant (Figure 1)) [6]. Two general mechanisms exist for in vivo HI: parthenogenesis (a seed develops without fertilization) and uniparental genome elimination [13]. Specifically, in vivo methods for artificial HI include sparse pollination, pollen irradiation, parthenogenesis, seed selection with twin embryos, and wide hybridization [14,15]. During parthenogenesis, an embryo develops spontaneously from an unfertilized egg cell (reviewed in [16]). The endosperm formation can be autonomous or pseudogamous (without or with central cell fertilization, respectively), and the embryo develops through apomeiosis (in the absence of meiosis). Overall, this process is referred to as apomixis, i.e., clonal seed formation [17]. Apomixis is genetically controlled, with the most prominent example of the *PsASGR-BabyBoom-Like* gene from apomictic *Pennisetum* [18].

The first DH lines released were the cultivars Maris Haplona of rapeseed (*Brassica napus*) and Mingo in barley (*Hordeum vulgare*) in the 1970s and 1980s, respectively [19,20]. DH protocols have been developed for more than 200 plant species [14]. Another culture and wide hybridization are considered the most widespread techniques for generating DH lines in crops (Figure 1).

Depending on the responsiveness of the gametes to tissue culture, haploid plants can be derived from either male (microspores or pollen) or female (megaspores or ovules) haploid cells, thus being of either paternal or maternal origin, respectively [21]. Male HI involves culturing of undifferentiated male gametes or microspores, either as isolated cells in the medium (isolated microspore culture (IMC)) or directly through culturing the anthers [8]. The technique is used in *Triticeae* species (bread wheat, durum, triticale, barley, etc.), rice, tobacco, rapeseed, and pepper [22,23]. The anther culture is a relatively simple method with a good yield of haploid plants in large-scale anther culture operations [20]. An extension to the anther culture is a ‘shed microspore’ technique where anthers are stimulated to dehisce and release their microspores into the culture medium. Microspores exposed to stress have the potential to undergo androgenesis and form calli that can eventually produce green haploid plants. Isolated microspore culture (IMC) has an advantage over another culture since the wall does not surround the cells; there is direct access to the media components and treatment conditions. Under optimal conditions, IMC results in a higher number of haploid embryos than another culture. Additionally, haploid plants are exclusively derived from the preconditioned male gametes but not from diploid somatic mother tissue from the anther walls. At the same time, the technique is highly genotype-dependent and suffers from plastid instability that, depending on species, results in the production of a significant number of nonviable albino plants [20]. Nevertheless, IMC is an attractive biotechnology tool since it allows the generation of fully homozygous plants from single cells that can be edited using designed endonucleases [24,25]. Similarly, microspore culture is a valuable method for cell biology and allows the identification of various stressors that redirect the cells from the limited gametophytic to the totipotent sporophytic pathway [26]. Notably, one of the essential embryogenesis-related genes, *BABY BOOM* (*BBM*), was discovered in reprogrammed microspores [27]. Overexpression of either *BBM* alone or in combination with *WUSCEL* has been successfully used to break recalcitrance to somatic embryogenesis and plant regeneration in pepper (*Capsicum* spp.), maize (*Zea mays*), and other monocots [27,28,29,30]. 

In maize, three primary techniques used for HI are the generation of paternal haploids using *indeterminate gametophyte 1* (*ig1*) female mutant and pollination with either Stock6-derived lines or mutants for domain of unknown function 679 membrane protein (*dmp*) to generate maternal haploids [21,31]. The haploid induction rate (HIR) in the *ig1* mutants is around 3%. The mutation was first discovered in the inbred line Wisconsin-23 (W23), and the female mutant is used to produce paternal haploids. The resulting haploid plants contain the cytoplasm from the *ig1* female inducer line and genome from the male plant [21]. The *ig1* allele was mapped to a region encoding a LATERAL ORGAN BOUNDARIES (LOB) domain protein localized on chromosome 3 [32]. The LOB domain-containing proteins play a role in the lateral organ development in higher plants [33]. The second exon is disrupted in the W23 mutant line by insertion of a Hopscotch retrotransposon, causing abnormal embryo sac development [32]. At the same time, the exact mechanism of haploid induction in the *ig1* maize mutants remains unclear [21]. 

The Stock6-derived HI lines are used as the pollen donors and result in a maternal HIR of 2–3% [34]. Breeding efforts allowed the development of lines with combined HI alleles from Stock6 and W23 mutants having an HIR of around 7–16% [35,36]. Recently, the *MATRILINEAL* (*MTL*) gene mutation was identified as responsible for HI in the Stock6 maize line [37,38,39].

Paternal chromosome elimination can also be achieved through wide hybridization (Figure 1). One of the first reports demonstrating the applicability of such a technique was a ‘bulbosum’ method where cultivated barley, *Hordeum vulgare*, is pollinated with bulb barley (*H. bulbosum*) [40]. Following pollination, regular double fertilization results in the formation of a hybrid zygote and endosperm. Eventually, during cell division, paternal chromosomes are eliminated from the zygote resulting in haploid embryos. The embryos in such a technique must be isolated from immature grains and grown on a sterile medium since hybrid endosperm also suffers from chromosome elimination and aborts [41]. The wide hybridization technique is also efficient in wheat and is widely used in breeding programs. Several donor plants such as maize, sorghum, teosinte, and pearl millet (*Pennisetum glaucum*) can be used for wheat pollination [42,43,44]. Nevertheless, the most common pollen donor is maize; this was successfully used for HI in triticale (× *Triticosecale*) [45], rye (*Secale cereale*) [46], and oat (*Avena sativa*) [47]. Unlike the microspore DH technique, wide hybridization does not suffer from albinism and has little genotype dependency. The HI system using wide hybridization was also developed in potato (*Solanum tuberosum*) through crosses of tetraploid (2*n* = 4*x*) *S. tuberosum* with the diploid (2*n* = 2*x*) relative *Solanum phureja* [48]. Di-haploids having the gametic chromosome constitution are produced from an unfertilized egg. However, unlike in cereals, sperm nuclei can fuse with the central cell of the ovule to form a functional endosperm that supports embryo development [20].

Development of the egg cell into a haploid embryo in the absence of fertilization could be achieved through gynogenesis in onion (*Allium cepa*), sugar beet (*Beta vulgaris*), and some trees [49,50,51]. Additionally, it was possible to develop a method for in vitro regeneration of zygotes and early embryos in wheat through coculturing isolated wheat zygotes with embryogenic pollen [52]. The gynogenesis technique in onion and sugar beet requires a particular growth temperature for the donor plants, and culturing is done on induction followed by regeneration media. In trees, gynogenesis is achieved through pollination with irradiated pollen of relatives. Overall, the efficiency of gynogenesis is relatively low, although this technique remains the only alternative of HI for some plant species [20].

Today, the DH technique finds wide application in the breeding of crops to produce commercial hybrids. The abundance of molecular markers aids in marker-assisted selection (MAS) and genomic selection (GS) for rapid screening of the breeding population to select the best allele combinations [6]. For instance, DH combined with MAS reduces time and resources for trait introgression into an elite variety [20]. Following a cross of elite variety with the line carrying the desired allele, the F_1_ plants are backcrossed to the parental line several times to clear the donor genome through recombination and segregation while maintaining the target locus. The technique is time-consuming and laborious and can be significantly accelerated by applying the DH method as early as following the first backcross. The stage of DH application during the backcrosses largely depends on the efficiency of the DH method for the generation of fertile plants. Additionally, the advantage of using DH versus F_2_ populations in both marker-assisted recurrent selection (MARS) and GS was greater in maize when many QTLs control the trait and the population size is small [53]. 

Production of F_1_ hybrids in crops also largely depends on either inbred or DH lines combined with male sterility (MS) [54]. Previously, reverse breeding was suggested to directly generate parental inbred lines from any hybrid [55]. The technique has been validated in *Arabidopsis thaliana* [56]. It is a multistep process involving Landsberg (Ler-0) and Columbia erecta (Col-0) to develop an F_1_ hybrid. First, meiotic crossover in the F_1_ hybrid was repressed through downregulation of the *DMC1* gene involved in the crossover. Afterward, the haploid plants were generated using a centromere-mediated haploid inducer line. Plants that underwent spontaneous chromosome doubling were analyzed using single-nucleotide polymorphisms (SNPs) to confirm absence of recombination. Eventually, the F_1_ hybrids were restored through crossing of the complementing DH lines. It remains to be shown whether reverse breeding can be applied to crops; nevertheless, the availability of HI lines in maize, wheat, and rice [57,58,59] suggests that it is a matter of time when we see this technology used in other species.

DH lines have been used to establish chromosome maps in several species, including wheat, barley, rice, and rapeseed [9]. In combination with high-throughput genotyping platforms such as microarray, exome capture, or genotyping by sequencing (GBS), the DH system significantly accelerates the development of genetic maps. Trait mapping is of particular importance to breeders, and generating a segregating DH population from F1 hybrids for marker–trait association studies has become common in barley, where efficiency of DH production is relatively high [60]. Some of the genetic techniques that benefit from complete homozygosity offered by the DH populations are bulked segregant analysis (BSA) and targeting-induced local lesions in genomes (TILLING). The BSA technique compares individuals from different extremes of phenotypic spectra for a given trait to find genetic signatures common in one population but absent in another (e.g., single-nucleotide polymorphisms, structural variants) [61]. The technique heavily relies on phenotyping data and, with the DH lines, can be repeatedly tested even in the field over multiple seasons. Similarly, mutagenesis of plant material for forward or reverse genetic studies is best done on either inbred or DH lines to avoid false-positive lines due to segregation of the starting material [62]. Although seeds are usually used as a starting material for mutagenesis, microspores were proposed as an attractive alternative due to the possibility of creating a mutagenized DH population directly from single cells, thereby avoiding potential chimerism or heterozygosity by using seeds [63]. At the same time, when a recessive mutation is lethal in a homozygous state, the DH system will not be suitable for such genetic analysis [20]. 

## 3. Haploid Induction in Crops through Gene Editing

Over the last few years, several studies demonstrated the use of genes altering the efficiency of DH production in crops. For example, loss of the centromere histone H3 (CENH3) protein improves the efficiency of wide hybridization (Table 1 and Figure 1). Specifically, the absence of the CENH3 protein from chromosomes of *H. bulbosum* potentially results in its chromosome elimination following *H. vulgare* × *H. bulbosum* hybridization and development of haploid embryos [64]. CENH3 is a centromere-specific histone variant and is a part of nucleosomes that are specifically deposited at the centromere regions, which are DNA foundations of the kinetochore. CENH3 attaches DNA to spindle fibers to segregate chromosomes and chromatids into the daughter cells during meiosis and mitosis [13]. It has been further demonstrated that complementation of the null *cenh3* allele with those carrying structural variants or those producing altered or partially deleted *CENH3* forms can lead to HI [57]. This could potentially occur due to competition among different forms of CENH3 and eventual omission of the altered centromeres by the hypothetical surveillance mechanism for chromosome assembly [21,65,66]. A recent alternative hypothesis also suggests that incompatibility between parents in wide crosses could differ in centromere sizes [67]. For example, oat centromeres are larger than those in maize and hybrids of these crops eliminate chromosomes of maize. In rare events, maize chromosomes could be retained in hybrids, and their centromeres expand similarly to those observed in oats [68].

Editing of *TaCENH3α* in wheat resulted in HI rate of ~7%, although heterozygous lines with genotype (+/r, −/−, −/−) for sub-genomes A, B, and D, respectively, where “+” is wild type, “−” is mutated, and “r” is restored frameshift alleles, triggered higher HI as compared to the null homozygous lines [69]. The authors suggested that the frameshift mutation allele at the sub-genome A was required for efficient paternal HI.

Two significant QTLs for HI in maize, *qhir1* (*ggi1*) and *qhir8*, determine approximately 66% and 20% of the genetic variance for the haploid induction rate (HIR), respectively (Table 1). Whereas the causative allele for *qhir1* was identified as a 4 bp insertion at the carboxy (C)-terminal coding region of *MATRILINEAL* (*MTL*), also known as *Patatin-Like Phospholipase A* (*ZmPLA1*) or *NOT LIKE DAD* (*NLD*) [37,38,39], a single amino-acid substitution in the first predicted transmembrane domain of the DOMAIN OF UNKNOWN FUNCTION 679 membrane protein (DMP) was responsible for the *qhir8* allele [31]. HIR of the *mtl* deficient plants is higher than in the *dmp* mutants, 3% versus 0.3%, respectively. The combination of both alleles allows increasing HIR up to 10% [31,70]. 

The *MTL* gene codes for pollen-specific phospholipase, and its mutation leads to genomic instability of sperm nuclei [71] (Table 1). Fragments of paternal chromosomes found in aneuploids indicate that the paternal genome is delivered to the zygotes [72,73], but it is eventually eliminated due to instability. The Cas9/gRNA–mediated mutation of the gene in maize and rice led to the production of haploid grains at a frequency of 6–10% and 2–6%, respectively [59,74]. It was also possible to identify the ortholog of the *MTL* gene in wheat, and its editing demonstrated a dose-dependent effect on HIR [75]. Whereas the double-knockout of the *TaMTL* homeologs of the A and D genomes (*TaMTL*−4A and *TaMTL*−4D) resulted in HIR of 10%, complete null mutant demonstrated an HIR of up to 31.6%. Similarly, in another study, the knockout of the *TaPLA*−A and *TaPLA*−D copies resulted in an HIR from 5.9 to 15.7% [58]. Although the *MTL* HI system is functional in staple monocots (maize, wheat, and rice), no *MTL* orthologs have been identified in dicots. At the same time, the *DMP* genes are conserved in dicots, and double mutation of the *AtDMP8* and *AtDMP9* genes using Cas9/gRNA in *Arabidopsis* triggered HI in self-pollinated mutants at a rate of around 2% [76]. The genes are involved in gamete fusion during double fertilization with a more significant contribution toward egg–sperm cell fusion than the central cell–sperm fusion leading to the endosperm development [77,78]. Therefore, the formation of haploid embryos in the *dmp* mutants is paralleled by developing the sexual endosperm [76].

## 4. Gene Editing in Haploid Cells 

Successful editing in crops using either biolistic or *Agrobacterium tumefaciens*-mediated delivery systems has been reported by different groups [88,89,90,91]. At the same time, the frequency of edited plant recovery depends on optimized promoters to drive the expression of sgRNA and Cas9 in transformants. For instance, it has been shown that selecting appropriate promoters affects the targeting efficiency in *Arabidopsis*, rice, maize, and wheat [75,90,92]. Although the level of the gRNA expression was not a limiting factor determining editing efficiency in rice, it played an essential role in *Arabidopsis* [92]. Similarly, the editing rates of TaU3, TaU6, and OsU6a promoters used to drive the expression of sgRNA in wheat were 61.4%, 36.0%, and 21.6%, respectively [75].

Furthermore, the use of the ubiquitin promoter for expression of the Cas9 gene in *Arabidopsis*, rice, and maize led to higher editing efficiency than the CaMV35S promoter, potentially due to lower activity of the latter in the plant gametes [91,93]. Different groups examined the effect of gamete-specific expression of Cas9 on GE efficiency to address this issue. The expression of Cas9 under egg cell-specific promoter allowed generating triple mutants in *Arabidopsis* T_1_ generation [94]. Similarly, when the Cas9 expression was driven by the *dmc1* promoter active in meiocytes and calli, the editing efficiency in transgenic calli was 100%, and biallelic mutations were recovered in up to 66% of the regenerated maize seedlings [90]. Therefore, editing in haploid gametes is preferred for the high-throughput recovery of homozygous mutants without chimerism [90,91].

**Figure 2:**
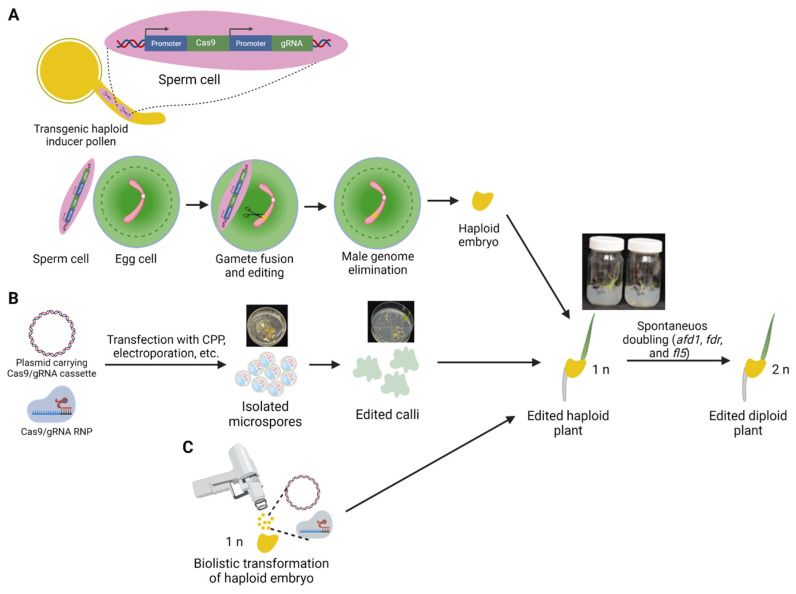
Gene editing in haploid tissues. (**A**) Editing of the maternal genome in maize or wheat using the Haploid Inducer-Mediated Genome Editing (IMGE) [95] or HI-Edit [96] methods through transient expression of the Cas9/gRNA cassette during pollination of non-inducer maize line with pollen from the HI line carrying the transgene. (**B**) Gene editing through transfection of isolated microspores using either the Cas9/gRNA DNA cassette or ribonucleoprotein (RNP) complex followed by regeneration of the edited haploid plants. (**C**) Direct gene editing in haploid tissues through biolistic delivery of the transgene or Cas9/gRNA RNP. Following the regeneration of haploid plants (*n*), genome doubling (2*n*) can be attained by manipulating selected genes through artificial or spontaneous doubling. *afd1*—*absence of first division1*, *fdr*—*first division restitution*, *fl5—formin-like-5*. Created with BioRender.com.

Recently, three independent groups reported developing simultaneous editing and HI in plants [95,96,97]. The method named Haploid Inducer-Mediated Genome Editing (IMGE) or HI-Edit relies on transient expression of the Cas9/gRNA cassette during pollination of a non-inducer maize line with pollen from the HI line carrying the transgene (Figure 2A). Due to selective elimination of the spermatid chromosomes following fertilization, no transgene transmission to the following generation occurs; therefore, haploid plants obtained in such a way carry only edits introduced by the Cas9 enzyme. Eventually, following chromosome doubling, homozygous edited DH lines are generated. If an elite line is used as a non-inducer pollen recipient, the method allows rapid transgene- and tissue culture-free introduction of edits into the elite background. This is essential since beneficial genetic modifications must be tested in elite varieties, often recalcitrant to genetic transformation. Kelliher et al. (2019) further demonstrated that the method could be applied to edit wheat genomes through the wide pollination with maize carrying the Cas9/gRNA transgene and even in *Arabidopsis* through using the *CENH3*-mediated HI [96]. The *Arabidopsis* HI line was obtained by swapping the native *AtCENH3* gene with a *ZmCENH3* transgene, which had no effect on the plant’s phenotype but resulted in 10% of androgenic haploids in crosses [98]. The *CENH3* HI system proved to be more efficient for generating edited haploid plants than the *MTL*-based method (3% editing rate in maize through the *MTL* induction versus 16.9% in *Arabidopsis* through the *CENH3*-mediated HI). The authors speculated that the small *Arabidopsis* genome potentially offers a higher editing rate for Cas9/gRNA than the complex maize genome [96]. Additionally, since, in the *CENH3* HI method, the transgene was expressed from the maternal genome, this potentially resulted in a higher amount of Cas9/gRNA produced during or after the fertilization stage combined with delayed paternal genome activation. In contrast, rapid uniparental genome elimination during the wide cross of wheat by maize pollen resulted in a lower editing rate in the haploid plants (1.8% for cultivar AC Nanda). The IMGE/HI-Edit technology may be applied in breeding elite crop varieties where the HI induction system was established. At the same time, it still suffers from some limitations since a specific gRNA transgene cassette needs to be transformed into an HI line for every new target. Additionally, although the method allows transgene-free GE to overcome regulatory oversight of transgenic plants, the same issue could be solved by segregating the Cas9/gRNA transgene in the following generation. Overall, the IMGE/HI-Edit technology adds to our understanding of the HI process by demonstrating that, prior to uniparental genome elimination, introduced transgenes can be expressed in the zygote [96] that can be used to generate higher-quality haploid grains.

An alternative approach to the IMGE/HI-Edit technology could be an in planta transformation of haploid embryos recovered from wheat × maize wide hybridization using bombardment (Figure 2C). Recently, Ryozo Imai’s group developed an in planta particle bombardment (iPB) method of wheat that omits culture-based transformation through the direct transformation of dissected shoot apical meristem (SAM) of mature embryos [99]. The technique allowed generating edited plants using Cas9/gRNA in model and elite Japanese wheat varieties [100]. Editing the *TaQsd1* gene (*quantitative trait locus on seed dormancy 1*) resulted in the recovery of homozygous mutants with a 1 week delay in the time required for 50% grain germination, thereby increasing tolerance to preharvest sprouting (grain germination on a spike). 

The iPB technique could find its extension by editing SAM in haploid embryos. Artificial or spontaneous chromosome doubling in the edited haploid plants would generate DH-edited F_2_ grains more quickly, thus accelerating the targeted introduction of desired mutations into elite cultivars. Furthermore, unlike the IMGE/HI–Edit technology, there is no need to create separate HI maize lines expressing the Cas9/gRNA cassette for every specific gene, thus allowing for rapid introduction of homozygous target mutations at different loci.

## 5. Haploid Microspores as a Potential Target for Gene Editing

Direct editing in haploid cells is possible by using isolated microspores primed for androgenesis and regeneration of haploid plants (Figure 2B) [24,25]. The microspore-based GE system in wheat has several advantages over methods based on somatic cells [25]. It provides many genetically identical and physiologically uniform embryogenic cells as targets for transformation, regeneration, and the early screening of GE events (Figure 2B). Millions of microspores can be isolated in a few minutes using a blender. In two recent studies, we demonstrated the potential of introducing target mutations using either Zinc finger nucleases (ZFNs) or Cas9/gRNA into isolated wheat microspore cells to generate edited haploid plants [24,25]. Transfection of microspores with the ZFN proteins using cell-penetrating peptides (CPP) facilitates DNA-free gene editing of the cells, eliminating the burden associated with transgene segregation and screening for the transgene-free mutant plants [24]. Unfortunately, the transfection process affects microspores viability; therefore, more resilient haploid embryos regenerated from the cells could also be used for transfection with the CPP–ZFN complex [24,101]. 

In another study, together with ThermoFisher Scientific (Waltham, MA, USA), we successfully delivered Cas9-dsRed plasmid DNA into wheat microspores using the Neon electroporation system. We optimized several factors that affect the delivery of Cas9/gRNA components into microspores. At the optimal potential difference of 500 V (pulsing), the efficiency of wheat microspore transfection using the Neon transfection system tended to be highest (2.2%) when 10–20 µg of plasmid DNA was delivered to 75,000 microspores. The microspores are immediately plated on glass-bottom Petri dishes containing 3 mL of NPB99 liquid medium to regenerate following electroporation. To promote embryoid development, three to four ovaries are added per plate, sealed with Parafilm, and incubated at 28 °C in the dark. After 20–30 days, embryos larger than 0.5 mm are removed from the Petri dishes, transferred to GEM culture medium, and exposed to 30 cm Sylvania Pentron 4100 K spectrum bulbs (21 W) delivering 125 μmol·m^−2^·s^−1^ lights (16 h photoperiod) at a constant 25 °C. Once the embryos turn green, they are aseptically transferred onto 50 mL of rooting medium in magenta vessels under the same conditions. Genomic DNA is then isolated for amplification with gene-specific primers. The presence of edits can be verified by various means (e.g., NGS, cleaved amplified polymorphic sequences assay, T7EI, and Sanger sequencing). Overall, the technology still suffers from a low transfection rate irrespective of the method used. Future improvement is needed for routing generation of the edited DH plants through the microspore culture. 

## 6. Application of Haploid Engineering for Hybrid Crop Production

A significant increase in crop yield can be achieved through heterosis in hybrid crops [54]. A prominent example is the production of hybrid crops in maize that resulted in a substantial boost in yield [54]. Similarly, heterosis could lead to up to 55% and 200% yield increase in rice and *Brassica* species, respectively [102]. A yield increase of up to 20% through the hybrid vigor could be obtained in wheat [103]. To achieve heterosis in a crop species, nearly completely homozygous parental lines must be created through either inbreeding or DH process. Compared to self-crossing, the DH technique significantly reduces the time and resources required to generate pure homozygous inbred lines [6]. In addition to the homozygous line, one of the parents should be MS to facilitate cross-pollination. Depending on the type of mutation, the MS mutants are divided into either cytoplasmic male sterility (CMS) or genic male sterility (GMS) (reviewed in [54]). Whereas the CMS mutants are deficient for the mitochondrial genes, the GMS mutants carry mutations in the nuclear genes. Both are considered three-line systems having an actual MS line, a restore line, and a maintainer line to produce hybrid grains and to maintain the MS line [104]. 

The development of pollen and anther is regulated by the targeting of many genes which can lead to MS in crops (reviewed in [54]). More than 40 GMS mutants are known for rice [105] and maize [106]. However, the polyploid nature of the wheat genome allowed the discovery of only 11 GMS genes [104,107]. Editing the *Ms26*/*CYP704* gene coding for cytochrome P450 monooxygenase involved in pollen exine synthesis resulted in the MS phenotype in maize, rice, sorghum, and wheat [108,109,110]. Similarly, editing of *Ms45*, the stamen-specific gene *Strictosidine synthase-like* (*SlSTR1*), or the *Ms1*-B homeolog in sub-genome B coding for a lipid-transfer protein that functions in pollen coat formation also resulted in MS in wheat [111,112]. In rice and maize, the MS traits are controlled by recessive genes, whereas, in wheat, a dominant gene *Ms2* was found in Chinese variety Taigu male-sterile wheat (TMSW) [113]. Until recently, the absence of a fertility restorer line hindered the deployment of the *Ms2*-based system for hybrid wheat production. It has been shown that all three *Ms2* homeologs are pseudogenes. However, the D-genome copy was reactivated due to the insertion of a terminal-repeat retrotransposon in a miniature element in the promoter region [114,115]. It was possible to completely restore male fertility through GE of the active *Ms2* copy [116], thereby helping to advance the *Ms2*-based hybrid breeding system for wheat.

The three-line systems are limited in the genetic resources of the restore and maintainer lines [117]. An alternative and more promising approach to the three-line MS is a conditional two-line breeding system that uses either photoperiod-sensitive genic male-sterile (PGMS) or thermosensitive genic male-sterile (TGMS) lines [117]. Restrictive and permissive conditions are used to either trigger or restore fertility, respectively. In photoperiod-sensitive lines of wheat, pollen abortion occurs mainly under long-day photoperiods [104]. For example, if the photoperiod is more than 14 h a day, the plants are cytoplasmically controlled male sterile and the MS lines are maintained by allowing selfing to occur with a photoperiod of less than 14 h [118,119,120]. It was possible to artificially engineer TGMS in different elite rice varieties by editing the *TMS5* gene that encodes the endonuclease RNase Z [117]. Conventional introgression of these null mutations into the elite wheat backgrounds through MAS is a laborious and lengthy process. The rapid introduction of mutations through GE holds promise to speed up the development of hybrid wheat lines with better adaptation to the changing climate and higher yields [2].

**Figure 3:**
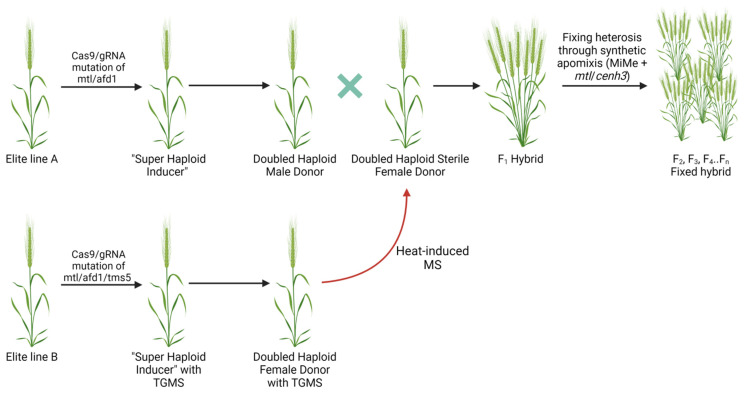
Hypothetical scheme for achieving and fixing heterosis by editing genes involved in haploid induction, spontaneous doubling, thermosensitive genic male sterility (TGMS), and mitosis instead of meiosis phenotype. First, selected elite lines A and B capable of generating the F_1_ progeny with hybrid vigor are converted into “super haploid inducer” lines through the *mtl*/*afd1* double mutation. ‘Super haploid inducers’ can generate haploids capable of producing fertile DH plants at a high rate through spontaneous chromosome doubling. The *tms5* mutation is also introduced in the genome of the female donor to facilitate heat-induced male sterility. The female DH sterile donor is pollinated with the male DH donor plant pollen to generate the F_1_ hybrid. Eventually, the hybrid vigor can theoretically be fixed through synthetic apomixes. *MTL*—*MATRILINEAL, afd1*—*absence of first division1*, *tms5*—gene involved in thermosensitive genic male sterility in rice, MiMe—mitosis instead of meiosis, MS—male sterility. Created with BioRender.com.

It is well established that heterosis cannot be propagated past the F_1_ hybrids. Fixing such phenotypes in the following generations would result in high cost and time benefits. It has been proposed that synthetic apomixes, when the offspring are generated asexually through seeds without meiosis or fertilization, can be used to fix the heterosis of the F_1_ hybrids [121,122]. One of the approaches to achieve artificial apomixes was to introduce mutations to three genes involved in meiosis (*spo11-1*/*osd1*/*rec8*) and replacing meiosis with mitosis-like division, a genotype termed MiMe (mitosis instead of meiosis, Figure 3) [123]. The mutant can generate clonal diploid gametes in *Arabidopsis* and rice. However, selfing of the MiMe plants doubles the ploidy in the progeny, and *CENH3*-mediated uniparental chromosome elimination is required to generate clonal diploid offspring [124].

Targeting a similar set of genes has been shown to induce true-breeding progeny through grains in the rice F_1_ hybrid variety [125]. Simultaneous editing of the *REC8*, *PAIR1*, *OSD1*, and *MTL* genes resulted in the generation of Fix plants with the same ploidy and heterozygous genotype as their parent. Nevertheless, the plants demonstrated reduced fertility due to the *MTL* mutation, and the effect was not fully penetrant. Further improvement to the technology will be needed for its routing implementation in plant breeding. Alternative *MTL* alleles or HI genes might be required to increase efficiency and eliminate the negative pleiotropic effect on the grain yield. The authors suggested that, due to the conservative nature of the studied genes, this technology may be applied to other commercial crops where GE is well established to develop the F_1_ hybrids [125].

## 7. Concluding Remarks and Future Perspectives

Efficient doubling of the genome in haploid plants still relies on highly toxic and potentially carcinogenic compounds such as colchicine, reducing the number of plants that withstand the treatment [126]. The use of alternative chemicals with lower cytotoxicity has also been reported (reviewed in [6]). At the same time, haploid plants can undergo spontaneous haploid genome doubling (SHGD) with different efficiency rates depending on the crop and cultivar [127]. For example, in bread wheat, it was possible to achieve chromosome doubling at the rate of 70%, whereas, in barley, it was as high as 90% [128,129]. Nevertheless, in our experience, the spontaneous doubling rate in wheat is significantly lower and is genotype-dependent. Other crops such as canola, rice, and rye demonstrate relatively high chromosome doubling rates (up to 40%, 60%, and 90%, respectively) [129,130]. Haploid fertility can be divided into haploid male fertility (HMF) and female fertility (HFF), and doubling of both cell lines is required for successful gamete formation and production of the DH lines [131]. In maize, the female fertility of haploid plants is relatively high. It has been shown that more than 90% of haploid ears produce grains without artificial doubling when pollinated with normal pollen from diploid plants [132]. At the same time, male fertility in haploid plants is strongly reduced and ranges from 2.8% to 46% [131]. Therefore, in the absence of induced chromosome doubling, HMF presents a bottleneck for the efficient production of the DH plants in maize. The effect in maize plants is genotype-dependent and demonstrates a genotype-by-environment interaction [133,134]. It was possible to detect QTLs controlling HMF and map the key QTL *qhmf4* to an ~800 kb region on chromosome 6 [131]. The most plausible candidate responsible for chromosome doubling was the *absence of first division1* (*afd1*) gene, a maize *rec8* homolog, affecting sister chromatid cohesion. The *afd1* mutant is compromised in the meiotic first division replaced by a single mitotic division, doubling the genome. Other studies in maize identified additional QTLs and gene mutants potentially involved in chromosome doubling in haploids such as *first division restitution* (*fdr*) and *formin-like-5* (*fl5*) [135,136,137,138]. Overall, genes involved in meiosis may regulate SHGD in *Arabidopsis* and maize [131,136,139].

Future work on improving GE in haploid plant tissues can include additional ways of transient manipulation of pathways involved in chromosome doubling or using particular mutants with a higher rate of SHGD. Orthologs of candidate genes identified in maize could potentially be explored in other crops. In most of the studies to date, mutations of the genes were responsible for haploid fertility [131,135,136]; therefore, their editing in important crops such as wheat, rice, and maize could potentially streamline production of spontaneous DH lines. Such an approach may lead to the development of ‘super haploid inducers’ with the ability to generate haploids capable of producing fertile DH plants at a high rate through spontaneous chromosome doubling [6]. We provide a hypothetical scheme of engineering DH donor lines for hybrid production and fixing the effect with the MiME genotype in the following generations (Figure 3). Although all key genes involved in HI, spontaneous doubling, conditional MS, and synthetic apomixes were recently described, practical implementation could still be challenging due to the negative collateral effect of some genes on seed yield (e.g., *mtl*, *cenh3*) [57,125]. Overall, the development of improved varieties in commercial polyploid crops will benefit from efficient implementation of GE in haploid tissues. The combined DH plus GE technology still suffers from limited throughput. It would be important to develop an approach to screen for plants that underwent SHGD following GE to prevent generation of DH lines with potentially a single allele edited. An early and strong expression of the Cas9/gRNA cassette in haploid tissues might be required to overcome this issue. Our increased understanding of promoter optimization to drive expression of the Cas9/gRNA cassettes, genes involved in HI, and spontaneous chromosome doubling will allow advancing translational genomics for accelerated trait improvement in commercial crops.

## Figures and Tables

**Table 1:** Genes involved in haploid induction in plants.

Gene Name	Gene Description	Plant	Haploid Induction Rate	Hi Engineering through GE	References
*Centromere histone H3* (*CENH3*)	Centromere-specific histone variant	*Arabidopsis thaliana*	Up to 34%	Yes	[65]
			Up to 44%		[66]
		*Brassica oleracea* var. *capitata*	Not tested	Yes	[79]
		*Cucumis melo*	1.50%	Not tested	[80]
		*Cucumis sativus*	1%	Not tested	[80]
		*Daucus carota*	Not tested	Yes	[81]
		*Hordeum vulgare*	0	Not tested	[82]
		*Oryza sativa*	1%	Not tested	[83]
		*Solanum lycopersicum*	2.30%	Not tested	[83]
		*Sorghum bicolor*	Not tested	Yes	[84]
		*Triticum aestivum*	~7%	Yes	[69]
		*Zea mays*	Up to 3.6%	Yes	[57,85]
*DOMAIN OF UNKNOWN FUNCTION 679 membrane protein* (*DMP*)	DMPs are involved in gamete fusion during double fertilization	*Arabidopsis thaliana*	~2.1% in *Atdmp8dmp9* double mutant	Yes	[76]
		*Zea mays*	0.30%	Yes	[31]
*indeterminate gametophyte1* (*ig1*)/*LATERAL ORGAN BOUNDARIES* (*LOB*)-domain protein	Involved in the lateral organ development in higher plants	*Zea mays*	3%	Not tested	[86]
*MATRILINEAL* (*MTL*)/*Patatin-Like Phospholipase A* (*ZmPLA1*)/*NOT LIKE DAD* (*NLD*)	Pollen-specific phospholipase	*Oryza sativa*	~6% in *Osmatl* background	Yes	[59]
		*Triticum aestivum*	Up to 15.7% in *tapla-a* and *tapla-d* double mutant	Yes	[58]
			31.6% in *TaMTL* triple mutant	Yes	[75]
			Not tested	C–to–T base editing	[87]
		*Zea mays*	3%	Yes	[37]

## Data Availability

Not applicable.
